# Phylogenetic Analysis of West Nile Virus Genome, Iran

**DOI:** 10.3201/eid2008.131321

**Published:** 2014-08

**Authors:** Nariman Shah-Hosseini, Sadegh Chinikar, Behroz Ataei, Anthony R. Fooks, Martin H. Groschup

**Affiliations:** Pasteur Institute of Iran, Tehran, Iran (N. Shah-Hosseini, S. Chinikar); Isfahan University of Medical Sciences, Isfahan, Iran (B. Ataei);; Veterinary Laboratories Agency, Addlestone, UK (A.R. Fooks);; Friedrich-Loeffler-Institut, Greifswald-Insel Riems, Germany (M.H. Groschup)

**Keywords:** West Nile virus, viruses, arbovirus, phylogenetic analysis, genome, lineage 2, encephalitis, Iran

**To the Editor:** West Nile virus (WNV) is a single-stranded, positive-sense RNA virus (≈11 kb) that is taxonomically classified within the family *Flaviviridae*, genus *Flavivirus*. WNV is found in Africa, Eurasia, Australia, and North America (*1*).

Comprehensive studies on phylogenetic relatedness of WNV strains have showed that WNV can be grouped into 5 lineages. Lineage 1 contains WNV strains from different regions, including northern, western, and central Africa; southern and eastern Europe; India; and the Middle East. Lineage 1 is subdivided into 3 clades. Clade 1A contains strains from Europe, northern Africa, the United States, and Israel, clade 1B contains Kunjin virus from Australia. Lineage 2 contains isolates from west, central, and eastern Africa and Madagascar. There is evidence that lineage 2 circulates in some regions of Europe (e.g., Italy, Austria, and Greece) (*2*,*3*). Lineage 3 contains Rabensburg virus 97–103, which was isolated in 1997 from *Culex pipiens* mosquitoes in South Moravia in the Czech Republic. Lineage 4 contains a new variant of WNV (strain LEIVKrnd88–190), which was isolated in 1998 from *Dermacentor marginatus* ticks in a valley in the northwestern Caucasus Mountains of Russia. Lineage 5 contains an WNV isolate from India (strain 804994) (*4*,*5*). In this study, we compared the phylogenetic relationships of WNV circulating in Iran to other WNV strains by using a partial WNV sequence isolated from an Iranian patient.

WNV was obtained from a blood sample from an Iranian patient who had encephalitis and was hospitalized in 2009 in Isfahan in the central highlands of Iran. The patient reported no history of animal contact, insect bites, blood transfusions, transplantations, and travel. He exhibited fever, headache, hypertension, and vomiting. On initial examination, he had a body temperature of 40°C. Laboratory investigations on the day of admission showed a leukocyte count of 240 cells/μL, a protein level of 52 mg/dL, and a glucose level of 50 mg/dL in a cerebrospinal fluid sample.

Further examinations were undertaken, and samples were sent to the Arboviruses and Viral Hemorrhagic Fevers Laboratory at Pasteur Institute of Iran in Teheran. For an IgG ELISA, wells in test plates were coated overnight with mouse hyperimmune ascitic fluid. Native antigen was added, and wells were incubated and washed. Test samples and peroxidase-labeled anti-human or anti-animal immunoglobulin were added. After incubation for 10 min, optical densities were read (*6*).

Viral RNA was extracted by using the QIAmp Viral RNA Mini Kit (QIAGEN, Hilden, Germany) from serum of the patient. A reverse transcription PCR was conducted by using a One-Step RT-PCR Kit (QIAGEN). Samples were subjected to 1 cycle at 50°C for 30 min to synthesize cDNA; 95°C for 15 min; and 95°C for 30 s, 54°C for 30 s, and 72°C for 60 s; and a final extension at 72°C for 5 min (*6*). The serum sample was positive for IgG against WNV. Molecular tests showed positive results for WNV.

The PCR product was sequenced by using the Big Dye Terminator V3.1 Cycle Sequencing Kit (Applied Biosystems, Foster City, CA, USA), the modified Sanger sequencing method, and an ABI Genetic Analyzer 3130 (Applied Biosystems) (*7*). Multiple alignments of nucleotide sequences were made by using ClustalW (http://www.clustal.org/). A phylogenetic tree was constructed by using 27 representative sequences of WNV and Japanese encephalitis virus (available in GenBank) and a 358-nt sequence of WNV (GenBank accession no. KJ486150), from the patient, which corresponds to nt 259–616 in the late region of the capsid gene and the early region of the membrane gene. Phylogenetic status of the sequence for the WNV strain from the patient was assessed by using the neighbor-joining algorithm in MEGA5 (*8*). Reliability of phylogenetic groupings was evaluated by using the bootstrap test (1,000 replications). Japanese encephalitis virus SA14 sequence was used as an outgroup in phylogenetic analysis of partial genome sequences (*8*).

The phylogenetic tree identified clustering of isolates in 5 lineages. Lineage 1 had 2 sublineage (clade 1A and clade 1B). All sequences in lineage 1 were geographically distinguishable. Clade 1A contained strains from Europe, Africa, the Middle East, and the United States; clade 1B included the Australian strain NSW 2011 (JN887352). The WNV sequence from Iran (KJ486150) was grouped into lineage 2 and had 99% identity with the 358-bp region of WNV strain ArB3573/82 from the Central African Republic. Although it was believed that lineage 2 strains circulate only in Africa, reports of their emergence primarily in Balkan countries (*9*) and in our study support the presence of a lineage WNV 2 strain in the Middle East, particularly in western Asia (Figure).

**Figure:**
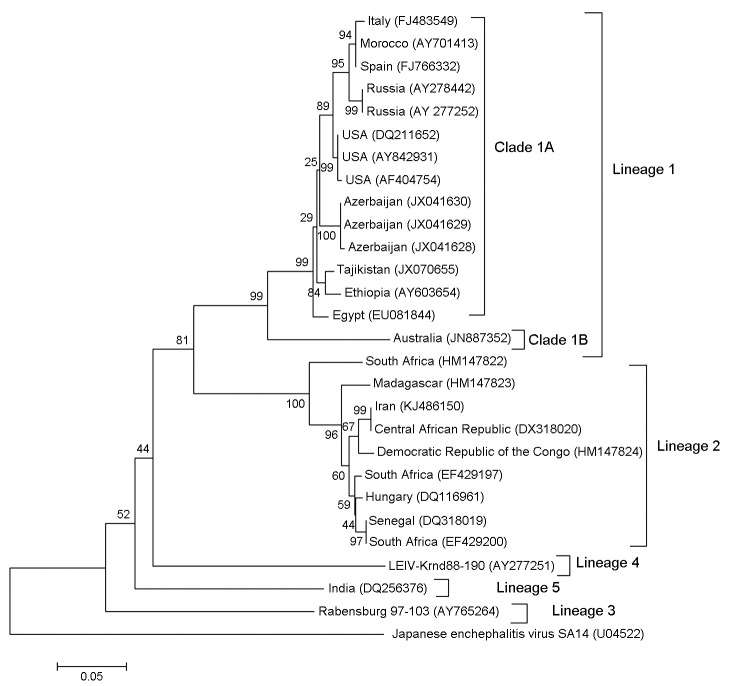
Phylogenetic tree based on a 358-nt sequence (nt 259–616) of 27 strains of West Nile virus (WNV) generated by using the neighbor-joining algorithm in MEGA5 (*8*). Japanese encephalitis virus was used as an outgroup. Location of virus isolation and GenBank accession numbers (in parentheses) are provided. The WNV sequence from Iran (KJ486150) was grouped into lineage 2 and had 99% identity with the 358-bp region of WNV strain ArB3573/82 from the Central African Republic. Scale bar indicates nucleotide substitutions per site.

Although the patient did not report any mosquito bites, this infection route cannot be excluded because he was a farmer and spent most of his time outdoors. Previous studies have demonstrated that nearly all human WNV infections were a consequence of mosquito bites (*10*). Our study should increase awareness of WNV infections as a public health threat. In future studies, priority should be given to investigations of the ecology, occurrence, and epidemiology of the different WNV strains circulating in Iran.
